# Hybrid cardiac rehabilitation in patients with durable left ventricular assist devices: the REHAB-ASSIST study

**DOI:** 10.3389/fcvm.2026.1932286

**Published:** 2026-09-03

**Authors:** Ramón Garrido-González, Elisa Velasco Valdazo, Mónica Barbero Campos, Paloma Remior Pérez, Alba Martín Centellas, Cristina Daniela Mitroi, Francisco José Hernández-Pérez, Manuel Gómez Bueno, Clemencia de Rueda Panadero, Marta Jiménez-Blanco Bravo, Susana del Prado Díaz, Susana Villar García, Javier Segovia Cubero, José Luis Zamorano Gómez, Jesús Álvarez-García, Carmen De Pablo Zarzosa, Mercedes Rivas-Lasarte

**Affiliations:** 1 Cardiology Department, Puerta de Hierro University Hospital, Majadahonda, Madrid, Spain; 2 Biomedical Research Networking Center for Cardiovascular Diseases (CIBER-CV), Madrid, Spain; 3 Cardiology Department, Ramón y Cajal University Hospital, Madrid, Spain; 4 Physiotherapy, Cardiac Rehabilitation Unit, Ramón y Cajal University Hospital, Madrid, Spain; 5 Cardiac Surgery Department, Puerta de Hierro University Hospital, Majadahonda, Madrid, Spain

**Keywords:** 6 min walk distance (6MWD), advanced heart failure (AHF), cardiac rehabilitation (CR), cardiopulmonary exercise test (CPET), Kansas city cardiomyopathy questionnaire (KCCQ), left ventricular assist device (LVAD), telemedicine

## Abstract

**Purpose:**

Patients with durable left ventricular assist devices (LVADs) frequently remain functionally limited despite technological advances. Cardiac rehabilitation may improve functional status, yet access is limited, and hybrid models have not been evaluated. The REHAB-ASSIST study assessed the clinical outcomes, safety, and feasibility of a hybrid cardiac rehabilitation program in this population.

**Methods:**

In this prospective multicenter proof-of-concept study, LVAD recipients underwent a 12-week hybrid cardiac rehabilitation program combining supervised and telemedicine-supported home-based sessions. The primary clinical endpoint was change in peak oxygen uptake. Secondary endpoints included 6-minute walk distance and quality of life (Kansas City Cardiomyopathy Questionnaire). Safety and feasibility were assessed as separate prespecified domains. Exploratory analyses were performed at 6 months.

**Results:**

Fifteen patients were enrolled and thirteen completed the program. Median age was 66 years and 93% were male; all had a HeartMate 3® LVAD. Peak oxygen uptake increased modestly but did not reach statistical significance (11.2 vs 12.2 mL/kg/min, *p* = 0.11). Statistically significant and clinically meaningful improvements were observed in 6-minute walk distance (444 vs. 492 m, *p* = 0.028) and Kansas City Cardiomyopathy Questionnaire (75.1 vs. 82.2 points, *p* = 0.042). Ventilatory oscillations were significantly reduced (38.5% vs. 7.7%, *p* = 0.045). No severe device-related or exercise-related adverse events occurred during the program.

**Conclusions:**

In patients with LVADs, a hybrid CR program was feasible, with high center-based adherence and no severe exercise-related adverse events observed, and it was associated with meaningful improvements in 6-minute walk distance and quality of life. These findings suggest that submaximal and patient-centered endpoints may be more appropriate than peak oxygen uptake to evaluate rehabilitation in this population, and require confirmation in randomized trials.

## 1 Introduction

Despite advances in the treatment of heart failure (HF) (1, 2), up to 10% of patients develop advanced-stage HF, refractory to conventional therapies and associated with a one-year morbidity and mortality exceeding 50% (3). Durable left ventricular assist devices (LVADs) have emerged as an established therapeutic option and their use has increased substantially in recent years, driven by technological advances and improved outcomes, with a reduction in device-related complications (4–6).

However, LVAD therapy remains associated with significant limitations that impair patient functional capacity following implantation (7). Some of these limitations are inherent to the current device design, including the presence of a driveline externalized through the abdominal wall and connected to a controller and two batteries, requiring the patient to remain permanently connected. Other limitations are related to LVAD hemodynamics. The device is programmed at a fixed speed and provides continuous flow.

Variations in flow depend on preload and afterload, which in turn are influenced by a right ventricle that is not supported by the device. Finally, patient-related factors, such as physical deconditioning following advanced HF and major cardiac surgery further contribute to reduced exercise capacity (7). In this context, cardiac rehabilitation (CR) has been proposed as a key intervention that may help to improve some of these factors, although evidence supporting its effectiveness in this specific patient population remains limited. Most available studies are single-center, small, and non-randomized. They include different LVAD models, such as pulsatile devices and earlier continuous axial-flow pumps. These devices may behave differently during exercise compared with the current HeartMate 3®, a centrifugal-flow, magnetically levitated pump.

Despite these limitations, most studies report benefits of CR in terms of functional capacity and quality of life (8–19), and major international societies acknowledge the need for CR programs following LVAD implantation (14, 20). Nevertheless, in real-world practice, a minority of centers currently offer these programs to patients with LVADs. Several factors contribute to this gap. These include difficulties for patients in attending CR programs regularly, as well as logistical challenges for rehabilitation units in delivering appropriately specialized care.

In addition, the potential of telemedicine-supported CR to improve access and scalability remains largely unexplored. A hybrid model combining center-based supervised sessions with telemedicine-supported home-based training may represent a pragmatic solution to overcome these limitations. However, to date, no studies have evaluated such an approach in patients with durable LVADs. Therefore, the REHAB-ASSIST was designed as a proof-of-concept study to evaluate the clinical outcomes, safety and feasibility of a hybrid CR program combining supervised and remote sessions guided by a telemedicine application in patients with a HeartMate 3® LVAD.

## 2 Methods

### 2.1 Study design

Prospective, multicenter proof-of-concept study conducted at two tertiary care hospitals with experience in LVAD management and cardiac rehabilitation (CR): Hospital Puerta de Hierro, and Hospital Ramón y Cajal (Madrid, Spain)*.*

### 2.2 Study population

From March 2023 to April 2025, all patients aged ≥18 years with a HeartMate 3® LVAD were screened for inclusion. Patients were excluded if participation in a center-based supervised program was not feasible due to logistical reasons (mainly distance to the center) or if they were unable to perform cardiopulmonary exercise testing.

### 2.3 Objectives

Given the proof-of-concept design and limited sample size, outcome analyses were considered exploratory and hypothesis-generating.

#### 2.3.1 Clinical endpoints

The primary clinical endpoint was to evaluate the change in peak oxygen uptake (peak VO₂) assessed by treadmill cardiopulmonary exercise testing (CPX), from baseline to 3 months (end of the exercise program).

Secondary clinical endpoints included changes from baseline to 3 months in: 1) distance covered in the 6-minute walk test (6MWT), 2) quality of life assessed using the Kansas City Cardiomyopathy Questionnaire (KCCQ), 3) additional CPX parameters: ventilatory oscillations, VE/VCO₂ slope, first ventilatory threshold, respiratory exchange ratio (RER), oxygen uptake efficiency slope (OUES), ventilatory equivalents, and peak heart rate; and 4) changes in laboratory biomarkers [NT-proBNP, carbohydrate antigen 125 (CA-125)], inflammatory markers (C-reactive protein, erythrocyte sedimentation rate), or nutritional/metabolic markers (HbA1c, lipid profile).

As exploratory analyses, all the above endpoints were also compared between baseline and 6 months after inclusion.

LVAD controller-derived parameters at rest and peak exercise were analyzed as exploratory physiological outcomes.

#### 2.3.2 Safety endpoint

The primary safety endpoint was the absence of a composite of LVAD-related complications occurring during exercise including device alarms, device malfunction, driveline-related complications, sustained ventricular arrhythmias, chest pain or acute coronary syndrome, symptomatic hypotension, syncope, or heart failure episodes. Events were classified as severe if they required hospital admission and/or discontinuation of the exercise program.

#### 2.3.3 Feasibility endpoint

Feasibility was assessed based on three aspects: 1) recruitment, described by the number of patients assessed for eligibility and ultimately enrolled, 2) adherence to center-based supervised sessions, and 3) the implementation of the home-based sessions supervised via telemedicine.

Adherence was defined as completion of at least 75% of scheduled center-based supervised sessions. The remote phase included telemedicine-based supervision of home-based sessions. Adherence to the home-based sessions was not prespecified as a feasibility outcome. The application records were used only to describe engagement with and implementation of the telemedicine-supported component. The implementation of the telemedicine-supported component was described using the number and type of home-based sessions documented in the mobile application.

Prespecified exploratory outcomes included smartwatch-derived daily step count and sleep duration during follow-up.

### 2.4 Intervention

**Figure 1:**
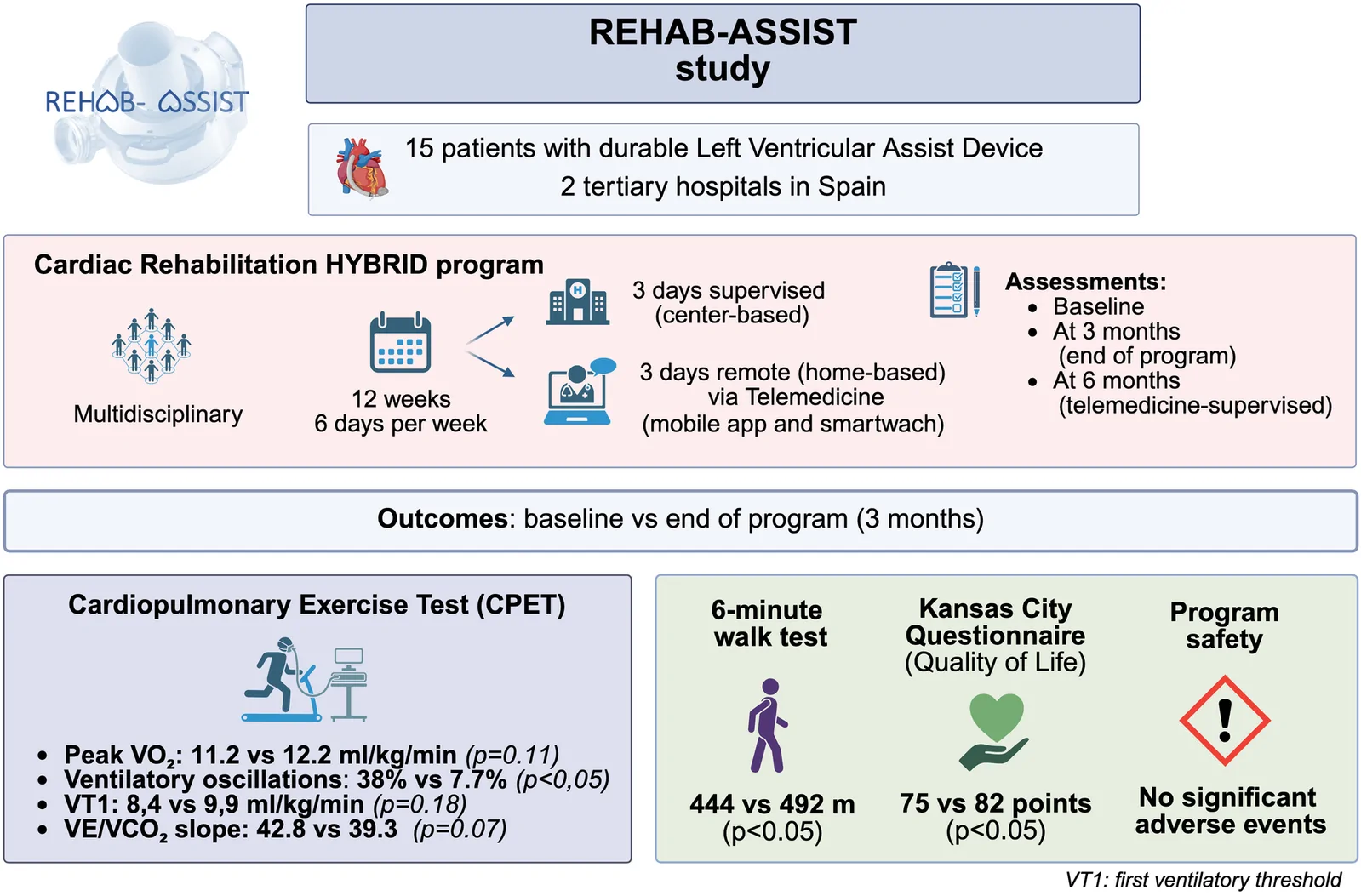
Central illustration of the multicenter REHAB-ASSIST study. Study protocol, sample size, and main and secondary outcomes. Created with BioRender.com. For quantitative variables, the paired Student's t-test was used. For qualitative variables, McNemar's test was applied for paired data. *VT1: first ventilatory threshold. Alternative text: Schematic overview of a multicenter proof-of-concept study including 15 patients with durable left ventricular assist devices recruited from two tertiary hospitals in Spain. The intervention consisted of a 12-week hybrid cardiac rehabilitation program delivered six days per week, combining three supervised center-based sessions and three home-based sessions via telemedicine using a mobile application and smartwatch. The program was multidisciplinary. Assessments were performed at baseline, at 3 months (end of program), and at 6 months (telemedicine follow-up). Clinical primary outcomes showed no significant change in peak oxygen consumption. Secondary clinical outcomes showed significant improvement in 6-minute walk distance and quality of life (Kansas City Questionnaire), significant improvement in ventilatory oscillations, and non-significant trends in ventilatory threshold and VE/VCO₂ slope. There were no significant adverse events reported.

Figure 1 (central illustration) summarizes the study protocol. A 12-week phase II CR program was developed using a hybrid model (center-based supervised and home-based sessions), with remote supervision via a telemedicine mobile application (Vitalera®) and a smartwatch (Fitbit®). After completion of the CR program, patients continued remote follow-up until 6 months from inclusion.

Three assessments were performed: baseline (inclusion), 3 months (end of the program), and 6 months (end of study). At each visit, a multidisciplinary evaluation was conducted, including physical examination, anthropometric measurements, blood tests, CPX, 6MWT, and LVAD controller data review. Health-related quality of life was assessed using the Kansas City Cardiomyopathy Questionnaire (KCCQ) at baseline and at 3 months (end of the program).

### 2.5 Cardiac rehabilitation sessions

Rehabilitation sessions were conducted at Hospital Ramón y Cajal. A total of 6 sessions per week were scheduled over 12 weeks: 3 center-based supervised sessions and 3 home-based sessions.

Center-based supervised sessions (75 min) included: 1) warm-up, 2) inspiratory muscle training using threshold devices, 3) continuous aerobic exercise on a treadmill or cycle ergometer, initially near the first ventilatory threshold, with progression to interval aerobic training approaching the second ventilatory threshold according to patient tolerance, and 4) resistance training for upper and lower limbs using weighted loads. Balance was assessed in all participants at baseline using the Tinetti Performance-Oriented Mobility Assessment. A score <25 was considered indicative of impaired balance and would have prompted the addition of specific balance exercises and repeat assessment at the end of the program.

Home-based sessions were performed 3 days per week and consisted of walking at an intensity close to the first ventilatory threshold (Borg scale 11–13), in two daily sessions of 20 min each (morning and afternoon), in addition to warm-up, respiratory muscle exercises, and resistance training for upper and lower limbs.

### 2.6 Sample size and statistical analysis

As reported in previous studies (11), a sample size of 15 patients was estimated to detect a 2.5 mL/kg/min difference in peak oxygen uptake, assuming an *α* level of 0.05 and a statistical power of 80%. Planned enrollment was not increased to account for potential attrition.

Data were collected using REDCap platform (Research Electronic Data Capture). Continuous variables are presented as mean (standard deviation) or median (interquartile range), depending on distribution, and categorical variables as percentages (absolute numbers). Comparisons between baseline and follow-up values were performed using paired data, using parametric or non-parametric tests as appropriate. The Shapiro–Wilk test was used to assess normality. A *p* value <0.05 was considered statistically significant. Statistical analyses were performed using Stata (StataCorp).

Feasibility outcomes were analyzed descriptively, without formal hypothesis testing. Screening and enrollment are reported as the number of patients assessed for eligibility, excluded, enrolled, initiating the intervention, and completing the program. Adherence to the center-based component is reported as the number and proportion of participants attending ≥75% of the 36 scheduled supervised sessions, together with the median (IQR) number of sessions attended. The telemedicine-supported component is described as the number and type of home-based sessions documented in the mobile application. Longitudinal smartwatch-derived data were analyzed using a generalized least squares model with random effects to account for within-subject correlation. Given the exploratory nature of the study, no adjustment for multiple comparisons was applied, and all *p* values should be interpreted as descriptive.

## 3 Results

### 3.1 Baseline characteristics

**Figure 2:**
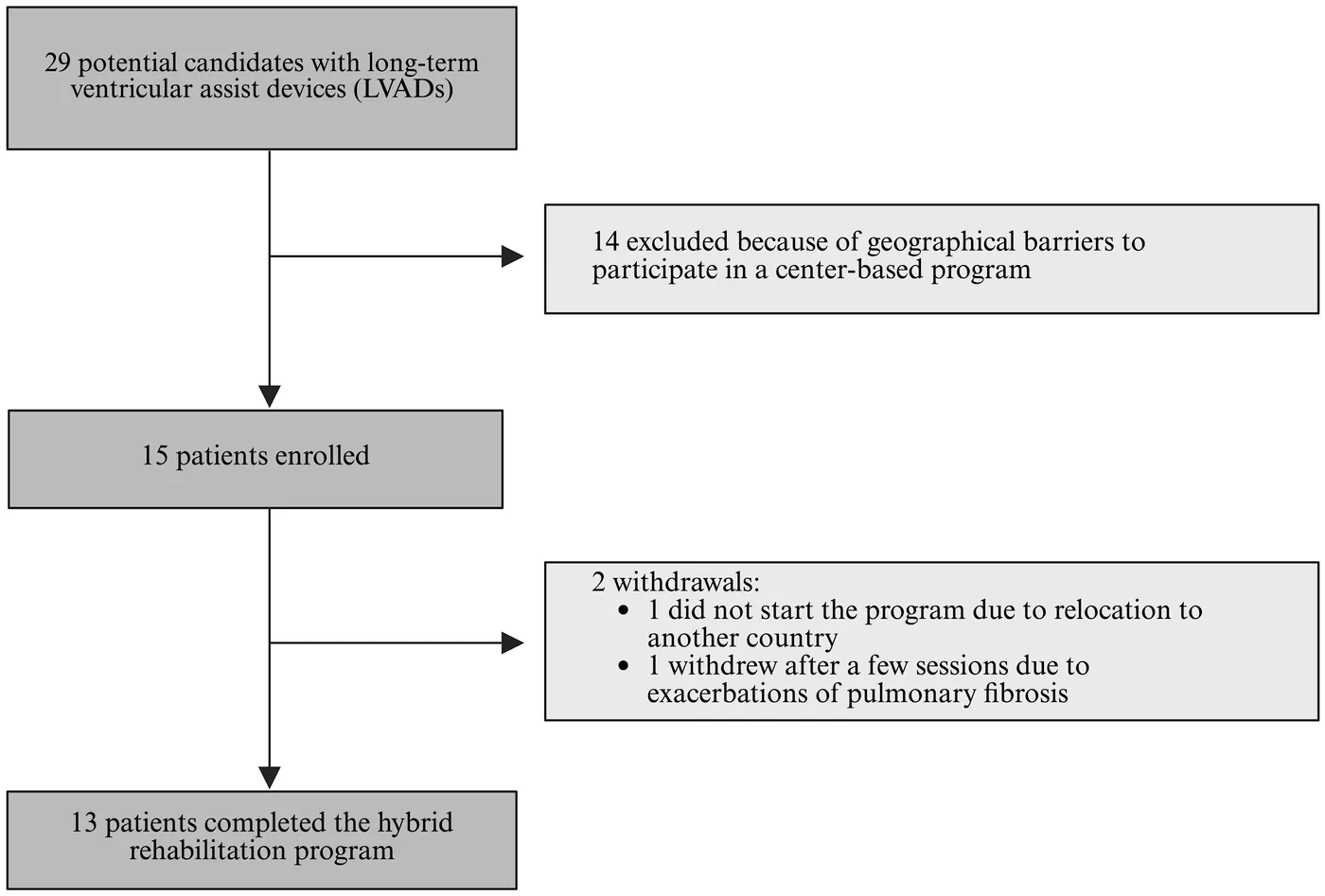
Study flow diagram. Created with BioRender.com. Alternative text: Flow diagram of patient selection and study participation. A total of 29 candidates with long-term ventricular assist devices were screened. Fourteen patients were excluded due to geographical barriers preventing participation in a center-based program. Fifteen patients were enrolled. Two patients withdrew: one due to relocation to another country before starting the program and one due to exacerbation of pre-existing pulmonary disease after a few sessions. Thirteen patients completed the hybrid cardiac rehabilitation program.

Between March 2023 and April 2025, a total of 15 patients were enrolled in the program (Figure 2). Of these, one patient withdrew after a few sessions due to exacerbation of pre-existing pulmonary disease, and another did not initiate the program due to relocation to another country.

**Figure 3:**
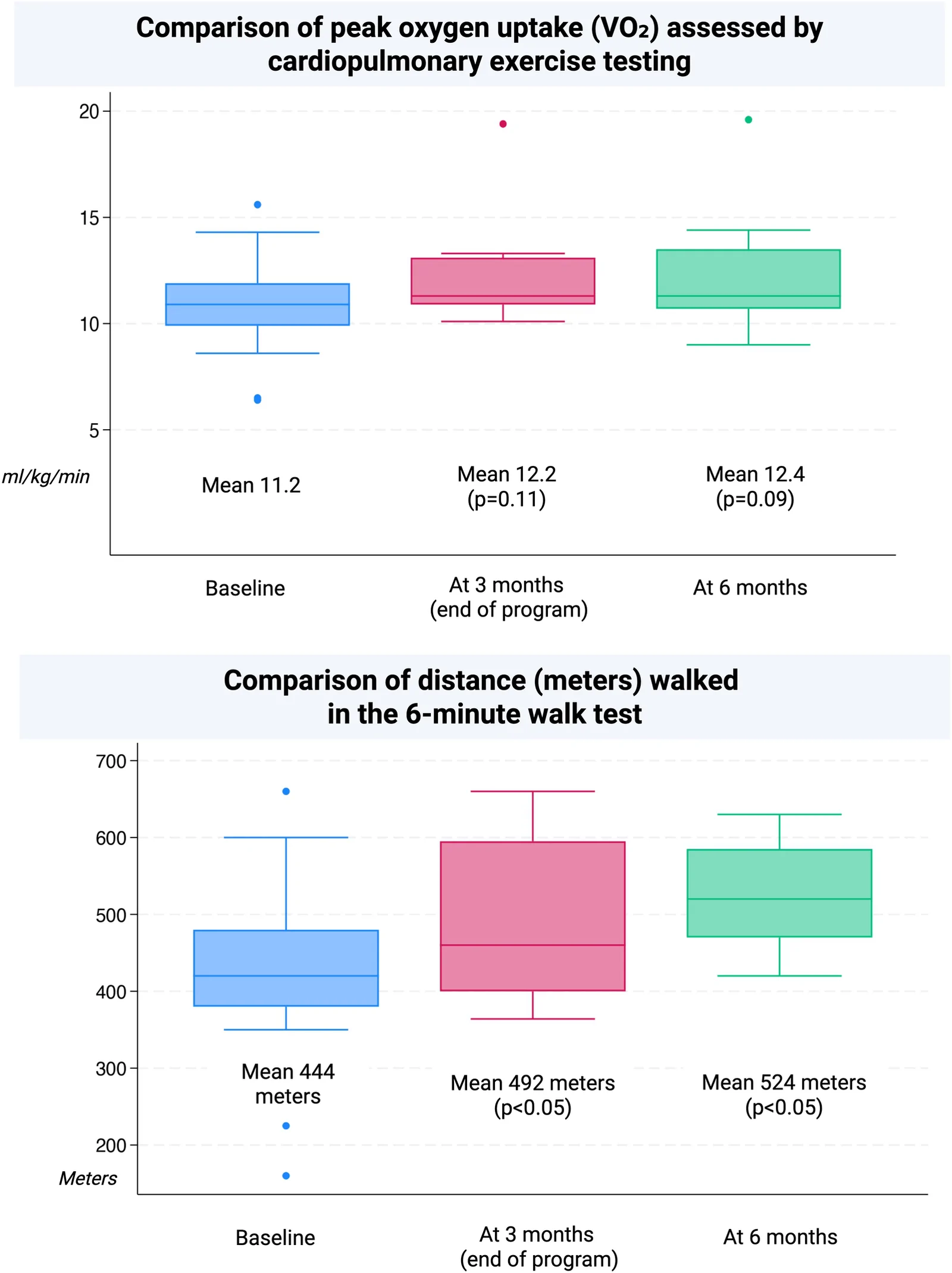
Box plots of peak oxygen consumption and 6-minute walk distance. For quantitative variables, the paired Student's t-test was used. Alternative text: Two sets of box plots comparing baseline, 3-month, and 6-month outcomes. The first set shows peak oxygen uptake (VO₂, mL/kg/min), with mean values of 11.2 at baseline, 12.2 at 3 months, and 12.4 at 6 months, without statistically significant differences. The second set shows 6-minute walk distance (meters), with mean values of 444 at baseline, 492 at 3 months, and 524 at 6 months, demonstrating a statistically significant improvement at follow-up. Each box plot displays median, interquartile range, and outliers.

Baseline characteristics are summarized in Table 1. The median age was 66 years [interquartile range (IQR) 60–73], and most patients were male (93%). All patients were White race (15); 14 were Spanish and 1 was German.

**Table 1:** Baseline characteristics of patients at inclusion.

Baseline characteristics of the 15 included patients	
Sex, male	14 (93%)
Age, yr	66 (60–73)
Race	15 (100%) White
Comorbidities	
Hypertension	10 (67%)
Diabetes mellitus	8 (53%)
Dyslipidemia	14 (93%)
Obesity (BMI ≥ 30)	5 (33%)
Chronic kidney disease (CKD-EPI <60 mL/min/1.73 m^2^)	7 (47%)
Glomerular filtration rate (CKD-EPI), mL/min	57 (50–68)
Chronic obstructive pulmonary disease (COPD)	4 (27%)
Prior stroke	4 (27%)
Significant peripheral artery disease	2 (13%)
Cardiac disease-related variables	
Ischemic dilated cardiomyopathy	10 (67%)
Non-ischemic dilated cardiomyopathy	4 (27%)
Valvular heart disease	1 (7%)
Atrial fibrillation	11 (73%)
LVEF, %	20 (15–25)
TAPSE, mm	12 (11–15)
NT-proBNP, pg/mL	1,617 (896–2,261)
ICD	13 (87%)
CRT	6 (40%)
Cardiac output and cardiac index estimated by thermodilution (median, IQR)	3.91 L/min (3.1–4.5), 1.93 L/min/m^2^ (1.7–2.3)
Durable ventricular assist device characteristics	
LVAD model	15 (100%) HeartMate 3®
LVAD therapy indication	
Destination therapy	12 (80%)
Bridge to transplant	2 (13%)
Bridge to decision/candidacy	1 (7%)
Duration of LVAD support, days	765 (120–1,332)
>1 year on LVAD support	8 (53%)
Anticoagulant therapy at study initiation	
Acenocoumarol monotherapy	7 (47%)
Acenocoumarol + acetylsalicylic acid	7 (47%)
Fondaparinux monotherapy	1 (6%)
Previous LVAD-related complications	
Prior gastrointestinal bleeding	2 (13%)
Cerebrovascular event	1 (7%)
Driveline infection	4 (27%)
Development of severe aortic regurgitation requiring TAVI	1 (7%)

Data are presented as n (%), median (interquartile range).

BMI, body mass index; COPD, chronic obstructive pulmonary disease; CRT, cardiac resynchronization therapy; ICD, implantable cardioverter-defibrillator; IQR, interquartile range; LVAD, left ventricular assist device; LVEF, left ventricular ejection fraction; TAPSE, tricuspid annular plane systolic excursion; TAVI, transcatheter aortic valve implantation.

All patients were supported with the HeartMate 3® device (15). The majority of LVADs (80%) were implanted as destination therapy. The median duration of LVAD support was 765 days (IQR 120–1332) at program initiation, being >1 year in 53% of patients. The most common underlying heart disease was ischemic cardiomyopathy (67%). Most patients (87%) had an implantable cardioverter-defibrillator (ICD).

There was a high burden of comorbidities among patients, the most frequent being diabetes (53%), chronic kidney disease (47%), chronic obstructive pulmonary disease (27%), and prior stroke (27%).

### 3.2 Clinical endpoints of the rehabilitation program

Clinical outcomes are summarized in Table 2 and Figures 1, 3. Regarding the primary endpoint, peak oxygen uptake increased by a mean of 1 mL/kg/min from baseline to the end of the exercise program (11.2 vs. 12.2 mL/kg/min), without reaching statistical significance (*p* = 0.11). A similar peak oxygen uptake value was observed at the 6-months follow-up visit, without reaching statistical significance (12.4 mL/kg/min, *p* = 0.09).

**Table 2:** Comparison of outcomes at baseline and at the end of the rehabilitation program (3 months).

	Baseline	End of program (3 Months)	Difference	*P* Value
CLINICAL OUTCOMES				
Cardiopulmonary exercise testing				
Peak oxygen uptake, mL/kg/min	11.2 (2.3)	12.2 (2.4)	**+1.0** (**2.2)**	***p*** **=** **0.11**
VE/VCO₂ slope	42.8 (8.5)	39.3 (6.3)	**−3.5** (**6.4)**	*p* = 0.07
RER	1.06 (0.13)	1.11 (0.15)	**+0.05** (**0.15)**	*p* = 0.31
First ventilatory threshold	8.4 (1.6)	9.89 (2.9)	**+1.45** (**2.73)**	*p* = 0.18
Ventilatory oscillations	5/13 (38.5%)	1/13 (7.7%)	**−30.8 percentage points**	***p*** **=** **0.045**
Test duration, minutes	6.39 (2.55)	7.39 (1.95)	**+0.99** (**1.94)**	*p* = 0.09
Peak heart rate, beats/min	113.9 (24.1)	129.3 (19.6)	**+15.3** (**19.3)**	***p*** **=** **0.019**
OUES	1.20 (0.33)	1.04 (0.24)	**−0.16 (0.44)**	*p* = 0.21
VECO₂, mL/min	39.5 (6.4)	39.3 (6.6)	**−0.1** (**3.2)**	*p* = 0.91
PETCO₂, mmHg	26.8 (3.1)	27 (3.3)	**+0.3** (**2.3)**	*p* = 0.76
6-minute walk test				
Distance, m	444 (124)	492 (109)	**+49** (**59)**	***p*** **=** **0.028**
Quality of life				
Kansas City Cardiomyopathy Questionnaire (KCCQ)	75.1 (17.3)	82.2 (14.8)	**+7.1** (**6.4)**	***p*** **=** **0.042**
PROGRAM SAFETY				
LVAD-related complications during exercise	1 low-flow alarm (hypertension)			
	0 arrhytdmias			
	0 dizziness or syncope			
	0 chest pain			
	0 driveline-related complications			
	0 heart failure episodes			

Data are presented as mean (standard deviation). HF, heart failure; KCCQ, Kansas city cardiomyopathy questionnaire; LVAD, long-term left ventricular assist device; OUES (oxygen uptake efficiency slope), PETCO₂, end-tidal CO₂ pressure; RER, respiratory exchange ratio; VECO₂, ventilatory equivalent for CO₂.

Bold values indicate the primary clinical outcome, absolute differences, and statistically significant results.

With respect to the secondary endpoints, a statistically significant improvement was observed in 6-minute walk distance (444 vs. 492 m, *p* = 0.028). The 6-minute walk distance was also significantly greater than baseline at 6 months (444 vs. 524 m, *p* = 0.003).

Analysis of further CPX parameters showed significant improvements in ventilatory oscillations (38.5% vs. 7.7%, *p* = 0.045) and peak heart rate increased from 113 to 129 beats/min (*p* = 0.019). Non-significant trends were observed for VE/VCO₂ slope (42.8 vs. 39.3, *p* = 0.07), first ventilatory threshold (8.44 vs. 9.89 mL/kg/min, *p* = 0.18), and respiratory exchange ratio (RER) (1.06 vs. 1.11, *p* = 0.31). No differences were observed in OUES or ventilatory equivalents.

At the end of the program, no statistically significant changes were observed in laboratory biomarkers [NT-proBNP, carbohydrate antigen 125 (CA-125)], inflammatory markers (C-reactive protein, erythrocyte sedimentation rate), or nutritional/metabolic markers (HbA1c, lipid profile) (Supplementary Table 1). Regarding controller-derived parameters, with similar pump speed, a significant increase in estimated flow was observed at peak exercise (4.2 vs. 4.6 L/min, *p* < 0.001), together with a slight increase in power (3.85 vs. 3.90 W, *p* = 0.039) and a significant decrease in Pulsatility Index (4.7 vs. 4.0, *p* = 0.014) (Table 3).

**Table 3:** Comparison of minicontroller parameters at rest and at peak exercise during cardiopulmonary exercise testing.

Parameter	At rest mean (SD)	At peak exercise mean (SD)	*P* value
Pump speed (rpm)	5,283 (142)	5,285 (150)	0.66
Estimated flow (L/min)	**4.2** (**0.45)**	**4.6** (**0.46)**	**<0**.**001**
Pulsatility index (PI)	**4.7** (**1.6)**	**4.0** (**1.6)**	**0**.**014**
Power (W)	**3.85** (**0.10)**	**3.90** (**0.10)**	**0**.**039**

Values are expressed as mean (SD). SD, standard deviation; rpm, revolutions per minute; PI, pulsatility index.

All participants had a baseline Tinetti score ≥25; therefore, no participant required the balance-specific training component or repeat Tinetti assessment.

### 3.3 Safety of the program

One participant did not met the safety composite endpoint because of low-flow alarms; however, no severe exercise-related adverse events occurred (Table 2). Those low-flow alarms were associated with hypertensive peaks, they were without clinical consequences, and resolved with adjustment of medical therapy without requiring discontinuation of the CR program.

### 3.4 Feasibility of the hybrid program

Between March 2023 and April 2025, 29 patients with a HeartMate 3® LVAD were screened (Figure 2). Fourteen patients were excluded because they lived outside the region (several hours from the rehabilitation center) and regular attendance at the center-based component was not considered feasible.

Finally, 15 patients were included in the program. Of these, one patient withdrew after a few sessions, and another did not initiate the program. All 13 patients who completed the program achieved the predefined adherence threshold of ≥75% of center-based supervised sessions (median 31 of 36 supervised sessions, IQR 29–35).

A total of 342 home-based sessions were documented through the mobile application during the full 6-month follow-up period among the 13 participants who completed the program. Each documented session represented a training episode including walking, with respiratory and/or resistance exercises recorded as additional components when performed. Accordingly, respiratory exercises were recorded in 327 sessions and resistance exercises in 270 sessions. Only one patient reported feeling unwell during two sessions due to “fatigue”; no other incidents were reported. Because the application could not distinguish between sessions that were not performed and sessions that were completed but not recorded, a formal adherence rate for the home-based component was not calculated.

**Figure 4:**
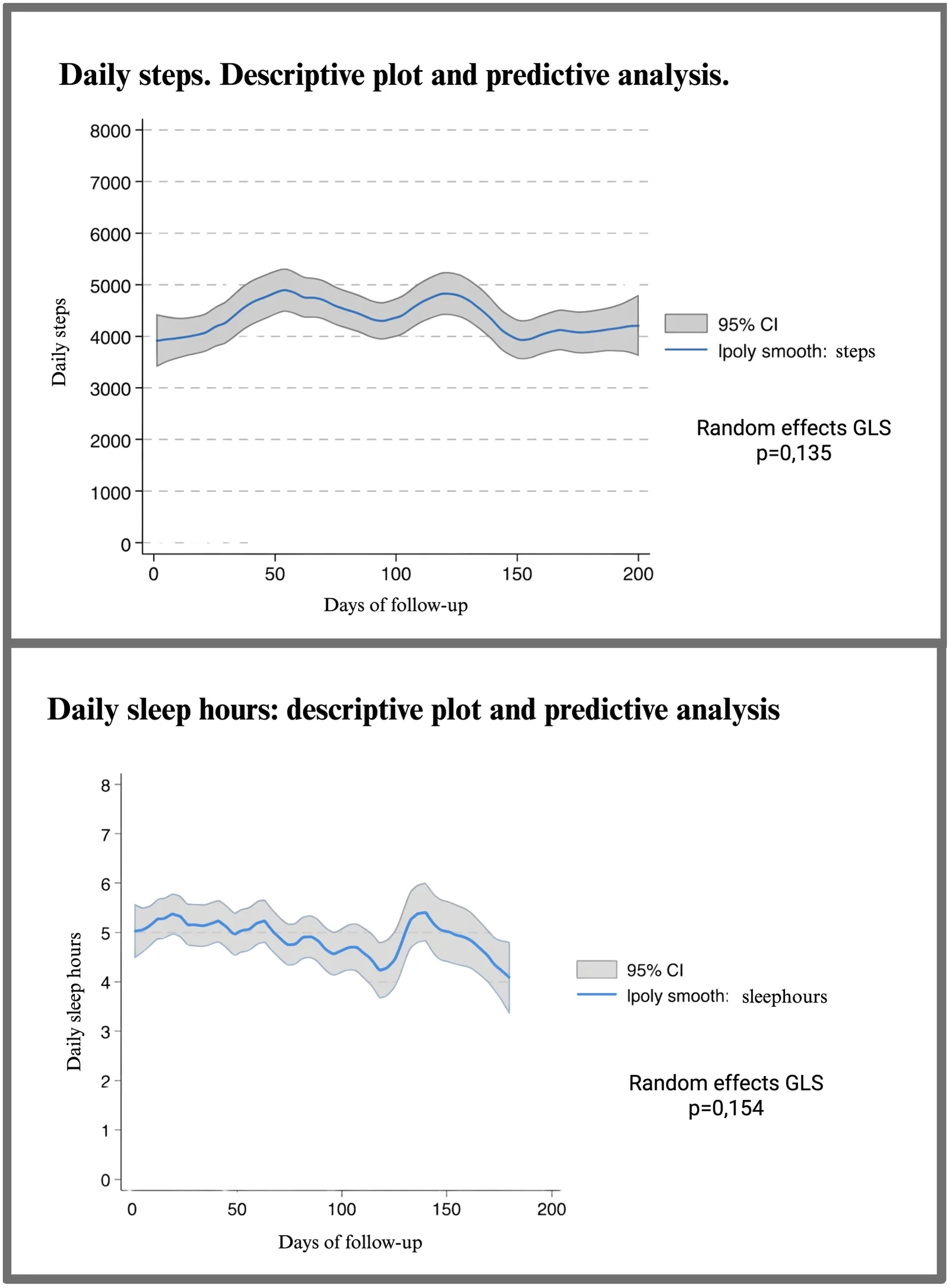
Data from the telemedicine application and smartwatch. A generalized least squares (GLS) model with random effects was used to account for within-subject correlation. Alternative text: Two longitudinal line graphs showing daily step count and daily sleep duration over approximately 200 days of follow-up. Each graph includes a smoothed trend line with a shaded 95% confidence interval. Daily step counts fluctuate around approximately 4,000–5,000 steps per day without a statistically significant trend over time (random-effects generalized least squares model *p* = 0.135). Daily sleep duration averages approximately 4 to 6 h per night, also without a statistically significant trend (*p* = 0.154). The analyses account for within-subject correlation using a random-effects model.

Analysis of smartwatch-derived data did not show significant changes in step count or sleep duration during follow-up (Figure 4).

## 4 Discussion

### 4.1 Main findings

The REHAB-ASSIST study provides novel evidence regarding the feasibility, safety, and potential clinical benefits of a hybrid cardiac rehabilitation program in patients with durable LVADs. To our knowledge, this is the first study to evaluate a hybrid model combining center-based supervised and remote exercise in this population.

Although the primary endpoint of peak oxygen uptake did not significantly improve, several clinically meaningful findings should be highlighted. First, functional capacity assessed by the 6-minute walk test improved significantly. Second, patient-reported quality of life, as measured by the KCCQ, showed a significant and clinically relevant increase. Third, no severe exercise-related adverse events were observed during the program, supporting a favorable preliminary safety profile. Collectively, these findings suggest the feasibility of implementing hybrid CR in this complex population, and indicate that the intervention was not associated with severe exercise-related adverse events, while providing preliminary signals of improvement in functional performance and patient-reported health status.

### 4.2 Hybrid cardiac rehabilitation as a solution to an unmet need

Despite guideline recommendations, CR remains underutilized in LVAD recipients, largely due to logistical barriers and the need for specialized multidisciplinary care (14, 20). In this context, hybrid models combining center-based and telemedicine-supported home-based interventions may represent a pragmatic and scalable solution.

This is the first study specifically evaluating such a hybrid approach in patients with durable LVADs. Importantly, approximately half of screened patients could not participate in a fully center-based program due to geographical constraints, highlighting a major gap between guideline recommendations and real-world feasibility. The present study directly addresses this gap, suggesting that a hybrid model can be successfully implemented with high center-based adherence and no severe exercise-related adverse events observed.

Furthermore, the global trend, including in Spain, is toward implantation predominantly as destination therapy, implying longer duration of support with devices that patients will carry throughout their lives (21, 22). Therefore, identifying strategies to improve functional status and quality of life in this population remains a priority, and CR may represent a key component in optimizing these costly therapies.

### 4.3 Peak oxygen uptake limitations and the relevance of submaximal and patient-centered endpoints

A non-significant mean increase of 1 mL/kg/min in peak oxygen uptake was observed at 3 months, which was also observed at 6 months. The absence of a statistically significant improvement in peak oxygen uptake should be interpreted in the context of the unique physiological characteristics of LVAD patients (7, 8, 11, 13). These patients frequently show submaximal exercise performance due to device-related hemodynamic limitations, including fixed pump speed, limited capacity to augment cardiac output, impaired right ventricular adaptation, and the constraints imposed by external pump components (7). As a result, peak oxygen uptake may underestimate clinically meaningful improvements in functional capacity.

LVAD recipients typically present a characteristic cardiopulmonary exercise profile, including ventilatory inefficiency, oscillatory ventilation, impaired chronotropic response, and an early or unidentifiable ventilatory threshold (7). In this context, submaximal parameters may be more informative. In our study, the marked reduction in ventilatory oscillations and the improvement in chronotropic response suggest enhanced ventilatory and cardiovascular adaptation to exercise. In addition, trends toward improvement in VE/VCO₂ slope, first ventilatory threshold, and respiratory exchange ratio, further suggest this physiological adaptation.

Importantly, these changes were accompanied by a 49-m improvement in 6-minute walk distance and a 7.1-point increase in KCCQ score. In patients with heart failure, changes of approximately 30 meters in 6-minute walk distance and 5 points in KCCQ score are commonly considered clinically meaningful (23, 24). Accordingly, both improvements exceeded these reference thresholds, supporting the clinical relevance of the observed gains in submaximal functional capacity and patient-reported health status. However, these thresholds were derived from broader heart failure populations and have not been specifically validated in LVAD recipients.

Controller interrogation findings suggest that the fixed-speed pump may respond passively to the hemodynamic changes of exercise. These observations should be interpreted cautiously, as flow and pulsatility index are controller-derived estimates rather than direct hemodynamic measurements. LVAD speed remained stable at its fixed setting, while estimated flow increased significantly and power showed only a minimal rise at peak exercise. This pattern may reflect exercise-induced increases in preload (driven by enhanced venous return through the skeletal-muscle pump and venoconstriction), a reduction in afterload (related to peripheral vasodilation), and an additional contribution from increased native right- and left-ventricular chronotropy and inotropy. The observed reduction in pulsatility index could reflect an increase in mean pump flow proportionally greater than the systolic–diastolic variation, potentially in the setting of reduced peripheral vascular resistance. However, these physiological interpretations cannot be confirmed from the present data.

These results suggest the concept that traditional maximal exercise parameters such as peak oxygen uptake may not represent the most appropriate primary endpoints in LVAD populations and highlight the potential value of functional and patient-centered outcomes in future studies.

### 4.4 Comparison with previous studies

Previous studies evaluating CR in LVAD recipients have been limited by small sample sizes, heterogeneous populations, and the inclusion of older-generation devices. Most have assessed exclusively center-based programs and included younger patients with shorter duration of LVAD support and a higher proportion of bridge-to-transplant indications (8, 10, 11, 19).

In contrast, the present study includes a homogeneous cohort exclusively supported with the HeartMate 3® device, predominantly in a destination therapy setting and with a relatively long duration of support. Despite these differences, the magnitude of improvement observed in functional capacity and quality of life is consistent with previous observational studies and meta-analyses, which have reported modest changes in peak oxygen uptake but more consistent improvements in submaximal performance and patient-reported outcomes (8, 11, 13). Regarding the 6-minute walk test, our findings are concordant with previous studies reporting improvements of 50–100 meters (8, 13, 15, 18, 25–28).

Notably, these findings are consistent with those of the Ex-VAD randomized trial, which reported a non-significant increase in peak oxygen uptake, along with improvements in functional capacity, 6-minute walk distance, and quality of life as assessed by the KCCQ (19).

### 4.5 Safety and feasibility of telemedicine-supported rehabilitation

A key finding of this study is that both supervised and remotely monitored exercise sessions appeared to be safe. No exercise-related severe adverse events were observed, even during home-based sessions supported by telemedicine. These findings extend previous evidence supporting the safety of center-based CR in LVAD patients (13, 17) and provide preliminary data supporting the feasibility and safety in a hybrid and partially remote setting.

The integration of wearable devices and telemonitoring enabled remote monitoring and may have contributed to both safety and adherence. These results are particularly relevant considering the substantial proportion of patients unable to access conventional CR programs due to geographical constraints. Despite incorporating a home-based component, however, the program still required regular attendance at three center-based sessions per week.

Because the intervention combined center-based supervised training with telemedicine-supported home exercise, the present study cannot determine the independent contribution of either component. The observed changes should be attributed to the hybrid program as a whole rather than to telemedicine alone.

Building on these findings, our group is currently conducting a multicenter randomized clinical trial to evaluate the efficacy and safety of a fully remote rehabilitation program compared with usual care, which currently consists of general exercise recommendations (RE-ACTION VAD trial, PI25/00394). This next step aims to further expand access to rehabilitation and to define the role of fully telemedicine-based strategies in this population.

### 4.6 Clinical implications and future directions

The present findings have important clinical implications. Hybrid CR programs may expand access to rehabilitation in LVAD patients, potentially reducing geographical and logistical barriers while providing preliminary evidence of feasibility and safety.

Future studies should focus on larger, randomized designs to confirm these findings and to better define optimal endpoints in this population. In particular, endpoint selection should prioritize functional performance and patient-reported outcomes over maximal exercise parameters. In addition, incorporating patient preferences, cost-effectiveness analyses, and long-term outcomes will be essential to guide the implementation of CR strategies in routine clinical practice.

### 4.7 Limitations

This study has several limitations. The small sample size, single arm, and non-randomized design limit the generalizability of the findings and preclude definitive conclusions regarding efficacy. Given the proof-of-concept design and limited sample size, clinical outcome analyses were considered exploratory and hypothesis-generating. The original sample size estimate did not include an allowance for attrition, and the loss of statistical power may have contributed to the lack of significance in the primary clinical outcome. Furthermore, multiple secondary and exploratory comparisons were performed without adjustment for multiplicity, increasing the possibility of type I error. Selection bias cannot be excluded, as nearly half of the screened patients could not participate due to geographical constraints. This may have selected participants with better geographical access, social support, or organizational capacity, further limiting generalizability to the broader LVAD population. Furthermore, the use of peak oxygen uptake as the primary endpoint may not fully capture clinically meaningful changes in this population with submaximal cardiopulmonary exercise test. The controller observations warrant a cautious interpretation, because they are not directly measured quantities but values derived by the controller from the motor-current signal and pump speed at an assumed blood viscosity. Finally, home-based adherence could not be reliably quantified because the application could not distinguish sessions that were not performed from those that were performed but not recorded.

Finally, there was a clear underrepresentation of female patients, reflecting the lower proportion of women with LVADs during the study period. Only one female patient was included across both centers. However, this sex imbalance has also been described in international registry data (21, 22).

## 5 Conclusions

A hybrid cardiac rehabilitation program combining center-based supervised sessions and telemedicine-supported home-based interventions was feasible, with high center-based adherence and no severe exercise-related adverse events observed in patients with LVADs. Although peak oxygen uptake did not improve significantly, the clinically meaningful improvements in 6-minute walk distance and KCCQ suggest that hybrid cardiac rehabilitation may enhance submaximal exercise performance and patient-reported quality of life. These findings suggest that hybrid CR may address important unmet needs in this population and support the use of functional and patient-centered endpoints to evaluate rehabilitation strategies in LVAD recipients. Larger randomized studies are warranted to confirm these findings and to further define the role of hybrid and fully remote rehabilitation strategies in LVAD recipients.

## Data Availability

The original contributions presented in the study are included in the article/Supplementary Material, further inquiries can be directed to the corresponding authors.
