# Supplementary material for: Hybrid cardiac rehabilitation in patients with durable left ventricular assist devices: the REHAB-ASSIST study
Source: Front Cardiovasc Med. 2026 Sep 3;13:1932286. doi: 10.3389/fcvm.2026.1932286 (PMC13582250; doi:10.3389/fcvm.2026.1932286)
Supplement: Supplementary file 1 [file Table1.docx]

**Supplementary Material**

**Supplementary Table 1. Changes in laboratory parameters between baseline and the end of the cardiac rehabilitation program (3 months).**

| **Laboratory parameter** | **Baseline** | **3 months** | **P value** |
| --- | --- | --- | --- |
| Creatinine, mg/dL | 1.24 (0.39) | 1.28 (0.37) | 0.56 |
| Estimated glomerular filtration rate (CKD-EPI), mL/min/1.73 m² | 61.0 (21.0) | 60.5 (20.0) | 0.87 |
| NT-proBNP, pg/mL | 1884 (1383) | 1703 (1027) | 0.44 |
| Carbohydrate antigen 125 (CA-125), U/mL | 35.3 (18.1) | 33.7 (28.9) | 0.81 |
| Total cholesterol, mg/dL | 122.6 (22.6) | 120.9 (20.7) | 0.67 |
| LDL cholesterol, mg/dL | 62.8 (19.2) | 59.8 (17.6) | 0.55 |
| HDL cholesterol, mg/dL | 39.1 (7.6) | 39.6 (5.6) | 0.76 |
| Glycated hemoglobin (HbA1c), % | 5.96 (0.67) | 5.98 (0.56) | 0.85 |
| C-reactive protein, mg/L | 12.0 (25.0) | 8.9 (18.1) | 0.23 |
| Erythrocyte sedimentation rate, mm/h | 18.5 (24.2) | 9.0 (8.8) | 0.23 |
| Hemoglobin, g/dL | 15.0 (2.1) | 15.0 (1.6) | 0.98 |
| Hematocrit, % | 45.8 (6.6) | 45.8 (5.5) | 0.99 |

*Data are presented as mean (standard deviation) and correspond to the 13 participants with paired baseline and 3-month assessments. Comparisons between baseline and 3-month values were performed on paired data using parametric or non-parametric tests as appropriate, as detailed in the Methods section.*

*Abbreviations: CA-125, carbohydrate antigen 125; CKD-EPI, Chronic Kidney Disease Epidemiology Collaboration; HbA1c, glycated hemoglobin; HDL, high-density lipoprotein; LDL, low-density lipoprotein; NT-proBNP, N-terminal pro–B-type natriuretic peptide; SD, standard deviation.*
